# Causal associations between disorders of lipoprotein metabolism and ten cardiovascular diseases

**DOI:** 10.3389/fcell.2022.1023006

**Published:** 2022-10-11

**Authors:** Qiannan Gao, Jiang-Shan Tan, Luyun Fan, Xiaoqi Wang, Lu Hua, Jun Cai

**Affiliations:** ^1^ Hypertension Center, FuWai Hospital, State Key Laboratory of Cardiovascular Disease, National Center for Cardiovascular Diseases, Peking Union Medical College, Chinese Academy of Medical Sciences, Beijing, China; ^2^ Center for Respiratory and Pulmonary Vascular Diseases, Department of Cardiology, National Clinical Research Center of Cardiovascular Diseases, State Key Laboratory of Cardiovascular Disease, Fuwai Hospital, National Center for Cardiovascular Diseases, Chinese Academy of Medical Sciences and Peking Union Medical College, Beijing, China

**Keywords:** cardiovascular diseases, causal associations, Mendelian randomization, lipoprotein, metabolism

## Abstract

Disorders of lipoprotein metabolism have been linked with an increased risk of cardiovascular diseases (CVDs) but the causal association is unclear. In this study, we investigated the causal association between disorders of lipoprotein metabolism and CVDs using two-sample Mendelian randomization (MR). The exposure was obtained from Finn genome-wide association studies (14,010 cases, 197,259 controls), and the corresponding CVDs were extracted from the largest published genome-wide association studies. A random-effects inverse-variance weighted method was used for the main analyses with a complementary analysis using the weighted median and MR-Egger approaches. Multiple sensitivity analyses were performed to assess horizontal pleiotropy. The MR analysis indicated positive associations of disorders of lipoprotein metabolism with coronary artery disease (odds ratio [OR] 1.670, 95% confidence interval [CI] 1.373–2.031; *p* < 0.001), aortic aneurysm (OR 1.394, 95% CI 1.199–1.619; *p* < 0.001), heart failure (OR 1.20, 95% CI 1.115–1.294; *p* < 0.001), hypertension (OR 1.011, 95% CI 1.006–1.091; *p* < 0.001), old myocardial infarction (OR 1.004, 95% CI 1.002–1.007; *p* = 0.001), and stroke (OR 1.002, 95% CI 1.001–1.003; *p* = 0.002). There is a suggestive causal relationship between disorders of lipoprotein metabolism and atrial fibrillation (OR 1.047, 95% CI 1.006–1.091; *p* = 0.026) and acute myocardial infarction (OR 1.003, 95% CI 1.001–1.005; *p* = 0.012). There was limited evidence of a causal association between disorders of lipoprotein metabolism and peripheral vascular disease and venous thromboembolism. Our findings indicate a significant causal association between disorders of lipoprotein metabolism and many CVDs, including coronary artery disease, aortic aneurysm, heart failure, hypertension, old myocardial infarction, and stroke. These associations may be useful for development of treatment strategies that regulate lipoprotein metabolism in patients with CVD.

## Introduction

Despite notable advances in prevention and treatment, cardiovascular diseases (CVDs) remain a leading cause of morbidity and mortality (Townsend et al., 2016). Increased cholesterol concentrations, smoking, hypertension, increased body mass index, diabetes, and physical inactivity are important risk factors for CVDs (Lim et al., 2012). In addition to these classical cardiovascular risk factors, a growing body of research has indicated that lipoprotein-related factors account for a considerable amount of residual cardiovascular risk (Chait et al., 2020). Lipoprotein(a) [Lp(a)], a complex of apolipoprotein(a) and a low-density lipoprotein-like particle, is a unique liver-derived lipoprotein (Koschinsky and Marcovina, 2004). The serum lipoprotein concentration is largely genetically determined and shows substantial interindividual variation. Numerous observational studies had demonstrated an association between a high lipoprotein concentration and increased risk of CVDs. The Copenhagen City Heart Study, which included 9,330 men and women who were followed up for 10 years, showed a stepwise increase in the risk of myocardial infarction (MI), with increasing Lp(a) and extreme Lp(a) levels predicting a 3–4-fold increase in risk of MI in the general population (Kamstrup et al., 2008). A meta-analysis of 126,634 participants in 36 prospective studies showed continuous, independent, and modest associations of Lp(a) concentration with the risks of coronary heart disease (CHD) and stroke under a wide range of conditions (Erqou et al., 2009). However, most of these findings are from correlational studies. Moreover, although an association between lipoprotein disorders and CVDs has been found in many observational studies, causality cannot be reliably inferred in view of potential confounders. Therefore, the causal association remains uncertain.

Mendelian randomization (MR) aims to overcome the limitations of conventional epidemiologic studies with respect to confounding and reverse causation (Liu et al., 2021). This method leverages the random assignment of genetic variants at gametogenesis, which are expected to be independent of confounding factors, to obtain causal estimates of exposure risks that are substantially less confounded and not susceptible to reverse causality (Richmond et al., 2016; Emdin et al., 2017). First, the germline mutation (not the somatic mutation) was used in the MR analysis. Therefore, the results were less influenced by the baseline demographic characteristics of different studies. Besides, the three assumptions of MR (Further details is provided in the Methods section) can further reduce the bias caused by the baseline demographic characteristics.

Previous genetic epidemiologic studies had implicated the Lp(a) level as a causal risk factor for CHD (Tsimikas, 2017). In one study, instrumental variable analysis showed that genetically elevated Lp(a) was associated with a hazard ratio of 1.22 (95% confidence interval [CI] 1.09–1.37) per doubling of the Lp(a) level (Kamstrup et al., 2009). Another study identified two Lp(a) variants (rs10455872, and rs3798220) that were strongly associated with both an increased lipoprotein level and an increased risk of CHD (Clarke et al., 2009). Beyond CHD, genetically lowered Lp(a) levels are associated with lower risks of peripheral vascular disease, stroke, heart failure, and aortic stenosis (Emdin et al., 2016; Schnitzler et al., 2019). However, Lp(a) are used as continuous variables in these studies and some potential associations may have been ignored. Therefore, we used the cut off value of lipoprotein metabolism as the categorical variable to explore the causal associations between the disorders of lipoprotein metabolism and CVDs. Besides, the risk of cardiovascular events might have been overestimated in the past given that Lp(a) levels and these events have been investigated in the same populations. Furthermore, the previous studies explored the relationship between the Lp(a) level and cardiovascular events; Lp(a) is a continuous variable, which may lead to underestimation of the association between Lp(a) and CVDs. Therefore, it is clinically important to include “disorder of lipoprotein” as a diagnosis when evaluating the relationship between disorders of lipoprotein metabolism and CVDs.

In this study, we sought causal relationships between disorders of lipoprotein metabolism and ten CVDs, including coronary artery disease, aortic aneurysm, heart failure, atrial fibrillation, hypertension, old MI, acute MI, stroke, peripheral vascular disease, and venous thromboembolism using two-sample MR analysis. The causality between disorders of lipoprotein metabolism and some of these CVDs suggests that regulation of lipoprotein might be a therapeutic target in patients with CVD.

## Methods

### Overall study design

**FIGURE 1:**
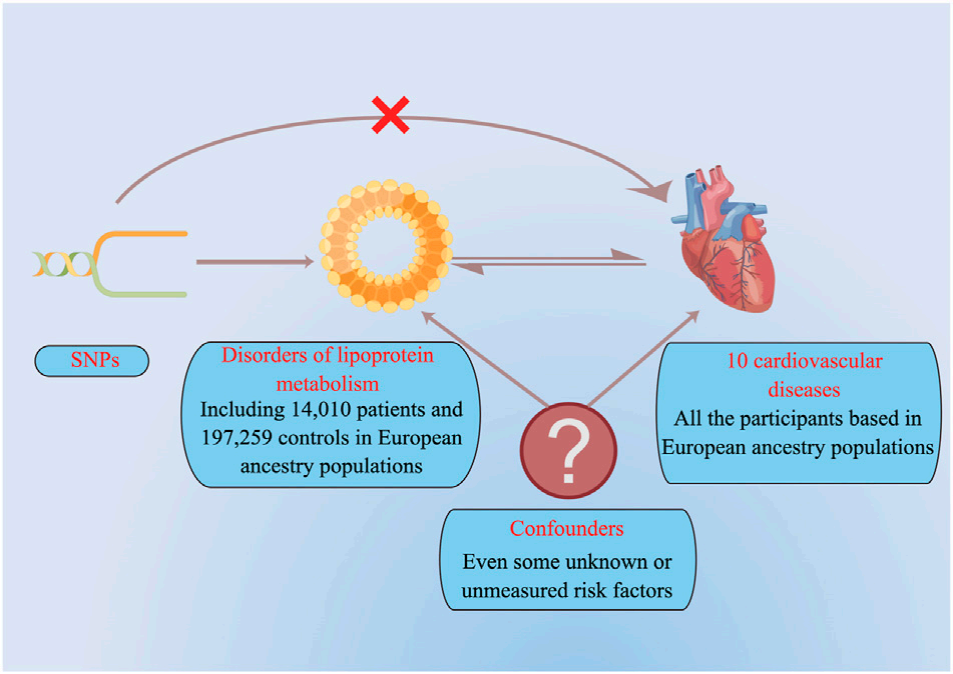
The study design of the present MR analysis.

Summary data were obtained from previously published studies, all of which had appropriate institutional review committee approval. Therefore, no additional ethical approval was required. We used two-sample MR (Lawlor, 2016) to explore the causal association between disorders of lipoprotein metabolism and the ten CVDs (Figure 1).

## Data sources

### Identification of SNPs associated with disorders of lipoprotein metabolism

From the most updated genome-wide association study (GWAS) on disorders of lipoprotein metabolism, that is, the Finn GWAS, we obtained 16,380,413 single nucleotide polymorphisms (SNPs) associated with disorders of lipoprotein metabolism identified from the primary meta-analysis of 211,269 European individuals, including 14,010 cases and 197,259 controls. Only SNPs that reached a genome-wide significant level (*p* < 5 × 10^–8^) in populations of European ancestry were used in this study. Independent variants are defined as low correlation (*r* ^2^<0.001) in the 1000 Genomes Project or HapMap22 data.

### Cardiovascular diseases

Summary statistics for ten CVDs (coronary artery disease, aortic aneurysm, heart failure, atrial fibrillation, hypertension, old MI, acute MI, stroke, peripheral vascular disease and venous thromboembolism) were obtained from the published GWAS (available at https://gwas.mrcieu.ac.uk/). Due to we used the summary data from previous published GWASs, the definitions of ten CVDs refer to the original definition in their GWASs without any modification. For each CVD, we used the most recent and largest GWAS summary statistics that were available to the public at the time of the analysis. Ultimately, 122,733 cases and 424,528 controls for coronary artery disease, 2,825 cases and 206,541 controls for aortic aneurysm, 47,309 cases and 930,014 controls for heart failure, 60,620 cases and 970,216 controls for atrial fibrillation, 54,358 cases and 408,652 controls for hypertension, 3,340 cases and 459,670 controls for old MI, 2,321 cases and 460,689 controls for acute MI, 7,055 cases and 454,825 controls for stroke, 1,456 cases and 461,554 controls for peripheral vascular disease, 4,620 cases and 356,574 controls for venous thromboembolism were included in this study. Detailed information on the CVDs included are shown in Table 1. There are three assumptions which must be satisfied in the MR analysis: First, the selected SNPs must be significantly associated with disorders of lipoprotein metabolism; Second, the selected SNPs must be independent of any other known risk factors; Third, the selected SNPs only influence the outcome through disorders of lipoprotein metabolism.

**TABLE 1:** Characteristics of selected GWASs.

Trials	Cases (N)	Controls (N)	Sample size (N)	Year of publication	No. of SNPs
Lipoprotein metabolism^a^	14,010	197,259	211,269	2021	16,380,413
Coronary artery disease (van der Harst and Verweij, 2018)	122,733	424,528	547,261	2017	7,934,254
Aortic aneurysm^a^	2,825	206,541	209,366	2021	16,380,417
Heart failure (Shah et al., 2020)	47,309	930,014	977,323	2020	7,773,021
Atrial fibrillation (Nielsen et al., 2018)	60,620	970,216	1,030,836	2018	33,519,037
Hypertension^b^	54,358	408,652	463,010	2018	9,851,867
Old myocardial infarction^b^	3,340	459,670	463,010	2018	9,851,867
Acute myocardial infarction^b^	2,321	460,689	463,010	2018	9,851,867
Stroke^b^	7,055	454,825	461,880	2018	9,851,867
Peripheral vascular disease^b^	1,456	461,554	463,010	2018	9,851,867
Venous thromboembolism^b^	4,620	356,574	361,194	2018	11,901,177

^a^ Output from the Finn GWAS.

^b^ Output from GWAS, pipeline using Phesant derived variables from UKBiobank.

GWAS, genome-wide association studies.

Genetic variants that passed uncorrelated (*r* ^2^ 169 < 0.001) SNPs associated with the risk factor at thresholds for a genome-wide level of statistical significance (*P* < 5×10^−8^ 170) were selected as instruments.

### Statistical analysis

In view of the lack of individual-level GWAS data, two-sample MR analysis was used to assess the causal association between disorders of lipoprotein metabolism and CVDs, as described previously (Tan et al., 2021a). Inverse-variance weighted (IVW) meta-analysis was used in the principal analyses to combine the instrumental variable-ratio estimates across the associated SNPs and account for correlations between genetic variants. We also used other established MR methods, including the weighted median and MR-Egger regression methods and MR-PRESSO (Pleiotropy Residual Sum and Outlier) for sensitivity analysis (Burgess and Thompson, 2015). The 95% CI for the odds ratio (OR) estimate was computed as the measure of effect size. These three methods (IVW, weighted median, and MR Egger) are based on different models of horizontal pleiotropy, and the consistence in these three different methods can make our results more reliable (Verbanck et al., 2018). If horizontal pleiotropy exists, the consistency between the corrected results of MR-PRESSO and IVW could ensure the reliability of our results. Finally, heterogeneous outcomes were detected using the modified Cochran Q statistic. All statistical tests were two-tailed. A Bonferroni-corrected threshold of *p* < 0.005 (a = 0.05/10 outcomes) was used. Associations with *p*-values between 0.005 and 0.05 were considered suggestive evidence of associations, requiring further confirmation. MR analyses were conducted using R version 4.0.3 (http://www.r-project.org) with the TwoSampleMR package.

## Results

### Genetic instrumental variables for disorders of lipoprotein metabolism and the ten CVDs

All genetic instruments associated with disorders of lipoprotein metabolism at a genome-wide significance level (*p* < 5 × 10^–8^) and with a corresponding effect on the ten CVDs are shown in the Supplementary materials.

### Effects of disorders of lipoprotein metabolism on the ten CVDs

**FIGURE 2:**
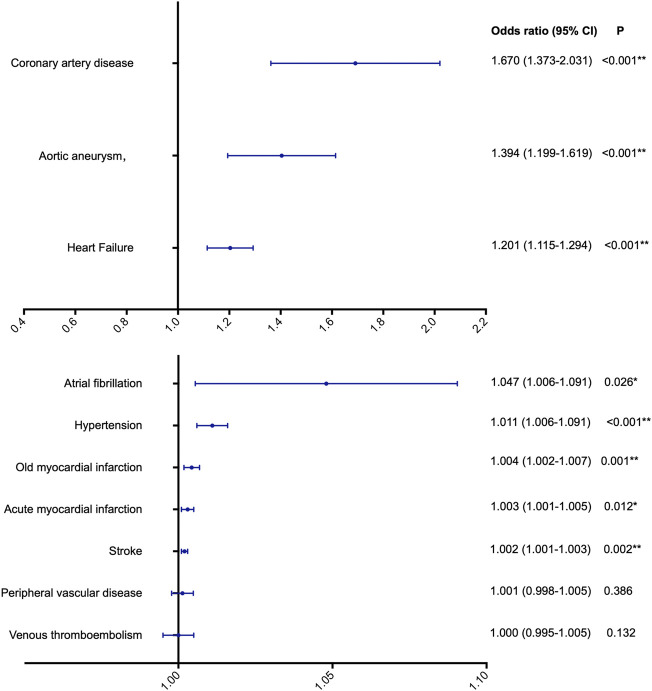
The potential causal association between lipoprotein metabolism and ten cardiovascular diseases. *p < 0.05, indicating a possible causal association; **p < 0.005, indicating a significant causal association.

Using IVW, we found evidence of causal associations between genetically predicted disorders of lipoprotein metabolism and most of the ten CVDs. Significant causal associations were found with coronary artery disease (OR 1.670, 95% CI 1.373–2.031; *p* < 0.001), aortic aneurysm (OR 1.394, 95% CI 1.199–1.619; *p* < 0.001), heart failure (OR 1.201, 95% CI 1.115–1.294; *p* < 0.001), hypertension (OR 1.011, 95% CI 1.006–1.091; *p* < 0.001), old MI (OR 1.004, 95% CI 1.002–1.007; *p* = 0.001), and stroke (OR 1.002, 95% CI 1.001–1.003; *p* = 0.002). There was a possible causal association with atrial fibrillation (OR 1.047, 95% CI 1.006–1.091; *p* = 0.026) and acute MI (OR 1.00, 95% CI 1.00–1.01; *p* = 0.012). However, there was no evidence supporting a causal association of disorders of lipoprotein metabolism with the risk of peripheral vascular disease or venous thromboembolism (Figure 2).

### Sensitivity analysis for our MR

Significant heterogeneity was found among the included studies for all CVDs other than stroke (Table 2). Fortunately, the sensitivity analysis in the weighted median and MR-Egger approaches showed similar estimation with IVW (Table 3). On further analysis, neither the MR-Egger intercept nor the MR-PRESSO detected any potential pleiotropy, indicating that the results of the primary analysis were robust and reliable (Table 2). These findings suggested that potential heterogeneity and directional pleiotropic effects did not influence the causal association between disorders of lipoprotein metabolism and any of the ten CVDs.

**TABLE 2:** The results of heterogeneity, horizontal pleiotropy test, and MR-PRESSO methods for cardiovascular diseases.

Outcome	*Heterogeneity test*		*Horizontal pleiotropy* ^a^		*MR-PRESSO*			
	Q	*P*	Or (95% CI)	*P*	Raw	*P*	Corrected	*P*
Coronary artery disease	825.316	<0.001	1.001 (0.937–1.069)	0.987	1.670 (1.373–2.031)	<0.001	1.599 (1.450–1.763)	<0.001
Aortic aneurysm	37.890	0.013	1.034 (0.985–1.084)	0.190	1.394 (1.199–1.619)	<0.001	-	-
Heart failure	100.129	<0.001	1.007 (0.982–1.033)	0.589	1.201 (1.115–1.294)	<0.001	1.213 (1.145–1.285)	<0.001
Atrial fibrillation	41.600	0.005	0.999 (0.997–1.001)	0.892	1.047 (1.006–1.091)	0.037	1.035 (0.998–1.074)	0.078
Hypertension	73.110	<0.001	0.999 (0.998–1.001)	0.468	1.011 (1.006–1.016)	<0.001	1.009 (1.004–1.013)	0.001
Old myocardial infarction	73.427	<0.001	1.000 (0.999–1.001)	0.848	1.004 (1.002–1.007)	0.008	1.002 (1.001–1.003)	0.001
Acute myocardial infarction	32.908	<0.001	1.000 (0.998–1.001)	0.661	1.003 (1.001–1.005)	0.036	1.001 (1.000–1.002)	0.112
Stroke	19.301	0.154	1.000 (0.999–1.000)	0.702	1.002 (1.001–1.003)	0.009	-	-
Peripheral vascular disease	39.818	<0.001	1.001 (0.998–1.003)	0.632	1.001 (0.998–1.005)	0.435	1.001 (0.999–1.003)	0.512
Venous thromboembolism	420.265	<0.001	1.000 (0.999–1.002)	0.623	1.000 (0.995–1.005)	0.939	0.999 (0.997–1.000)	0.061

CI, confidence interval; OR, odds ratio; WHR, Waist-to-hip ratio.

^a^ The MR-Egger intercept quantifies the effect of directional pleiotropy. *p* < 0.05 provides evidence that the exposure-associated single-nucleotide polymorphisms may influence the outcome through other pathways than through exposure.

**TABLE 3:** The results of sensitive analysis by the weighted median and MR Egger analysis.

Outcome	Weighted median analysis		MR egger analysis	
	Or (95% CI)	*P*	Or (95% CI)	*P*
Coronary artery disease	1.396 (1.310–1.487)	<0.001	1,665 (1.094–2.532)	0.029
Aortic aneurysm	1.388 (1.165–1.654)	<0.001	1.145 (0.831–1.577)	0.190
Heart failure	1.131 (1.058–1.210)	<0.001	1.153 (0.978–1.359)	0.105
Atrial fibrillation	1.017 (0.973–1.063)	0.449	1.053 (0.964–1.150)	0.264
Hypertension	1.011 (1.006–1.016)	<0.001	1.015 (1.004–1.026)	0.016
Old myocardial infarction	1.003 (1.001–1.004)	<0.001	1.003 (0.994–1.012)	0.494
Acute myocardial infarction	1.002 (1.000–1.004)	0.021	1.005 (0.993–1.018)	0.413
Stroke	1.002 (1.001–1.004)	0.005	1.003 (0.999–1.003)	0.512
Peripheral vascular disease	1.000 (0.998–1.002)	0.942	0.994 (0.968–1.021)	0.702
Venous thromboembolism	0.997 (0.996–0.999)	0.002	0.998 (0.988–1.008)	0.691

## Discussion

In this study, two-sample MR analysis revealed a causal association of genetically determined disorders of lipoprotein metabolism with increased risks of coronary artery disease, aortic aneurysm, heart failure, hypertension, old MI, and stroke in a population with European ancestry, a possible causal association with atrial fibrillation and acute MI, and no causal relationship with peripheral vascular disease.

In the past decade, disorders of lipoprotein metabolism have been linked to increased risk of CVDs. Large prospective general population studies have shown that high Lp(a) concentrations increase the risks of CHD (Kamstrup et al., 2008), nonfatal MI, and coronary death (Erqou et al., 2009). The large population-based Atherosclerosis Risk in Communities study, in which blacks and whites were followed for up to 20 years, showed a positive association between the Lp(a) level and cardiovascular events. The associations were at least as strong in blacks as in whites, but with a wider range of Lp(a) concentrations (Virani et al., 2012). In terms of pathophysiological mechanisms of action, *in vitro* or animal studies have implicated Lp(a) in key processes related to atherosclerosis, including formation of foam cells, proliferation of smooth muscle cells, and plaque inflammation and instability (Boffa et al., 2004; Ugovšek and Šebeštjen, 2021). Furthermore, Lp(a) and oxidized phospholipids drive valve calcification and disease progression in patients with aortic stenosis (Zheng et al., 2019). Nevertheless, residual or unmeasured confounding is of particular concern because these factors are not always considered or available in observational studies.

In recent years, large genetic epidemiologic studies have provided strong evidence of associations of high Lp(a) concentrations with increased risk of CVDs. Some genetic epidemiologic studies, including genome-wide association studies, have revealed the locus in the LPA gene, and some have specifically identified the rs3798220 SNP as being associated with an increased risk of coronary artery disease (Luke et al., 2007). Analysis of 17,576 potentially functional SNPs in three case-control studies of MI identified SNPs in the LPA gene that merit further examination for their potential association with MI (Shiffman et al., 2008). However, the associations observed between disorders of lipoprotein metabolism and CVDs in these studies do not confirm a causal relationship.

A strength of the present study is that we assessed the causal association between disorders of lipoprotein metabolism and ten CVDs using MR, which can provide strong genetic evidence of causality (Ference et al., 2021). MR refers to the random assortment of genes transferred from parent to offspring at the time of gamete formation. This method could aid observational epidemiology by potentially allowing an unbiased estimate of the effects of gene products on disease outcomes. Given that alleles are randomly assorted and fixed at conception, biases caused by confounding and reverse causality would not have been detected in our MR analysis. Our results represent the lifetime risk for CVDs due to elevated lipoprotein because genetic variation is stable throughout life. Furthermore, stratification by population can affect the findings of MR studies. However, we reduced this bias by using summary statistics data only for individuals of European ancestry.

Our analysis provided evidence that disorders of lipoprotein metabolism confer a higher OR for coronary artery disease, aortic aneurysm and heart failure. While the OR was small for hypertension, old MI and stroke, considering the high prevalence of these CVDs in population, a small increase in relative risk for high prevalence exposures can result in a large burden of disease. Our MR study can provide us clinical guidance to test and treat the elevated Lp(a) levels. Besides, it is important for us to consider its inclusion in global risk estimation based on our findings. Moreover, even though no causal association was observed for peripheral vascular disease and venous thromboembolism, the potential importance of a factor may exist within shorter time frames and further research is needed to investigate relevant discrepancies.

However, the study also has some limitations. First, only individuals of European ancestry were included in the analysis. In view of the variation in genetic characteristics of CVDs according to ethnicity (Tan et al., 2021b), our findings cannot be generalized to other ethnic groups. Second, individual-level data were not available. Therefore, we have been unable to provide a risk estimate adjusted for individual characteristics, such as age and sex. Third, it is still noteworthy that the association between “disorder of lipoprotein” and CVDs were less significant in the sensitivity analysis by MR Egger. But the results of IVW is the principle analysis and the other sensitivity analysis revealed similar estimation with IVW. Therefore, our MR results were reliable. Finally, although our MR analyses supported a causal relationship between exposures and outcomes, randomized cardiovascular outcome trials are needed to provide conclusive evidence of causality and to assess the potential clinical benefit of therapeutic strategies aimed at targeting disorders of lipoprotein metabolism.

In conclusion, using MR analysis, we have found potential evidence of a causal association between disorders of lipoprotein metabolism and CVDs, especially for coronary artery disease, aortic aneurysm, heart failure, hypertension, old MI, and stroke. Therefore, lipoprotein metabolism may be a target for prevention and treatment of CVD.

## Data Availability

The original contributions presented in the study are included in the article/Supplementary Material, further inquiries can be directed to the corresponding authors.
